# The Role of the Anesthesiologist during Magnetic Resonance-Guided Focused Ultrasound Thalamotomy for Tremor: A Single-Center Experience

**DOI:** 10.1155/2018/9764807

**Published:** 2018-07-05

**Authors:** Alon Sinai, Yeshayahu Katz, Menashe Zaaroor, Olga Sandler, Ilana Schlesinger

**Affiliations:** ^1^Department of Neurosurgery, Rambam Health Care Campus, Haifa, Israel; ^2^Department of Anesthesiology, Rambam Health Care Campus, Haifa, Israel; ^3^The Ruth and Bruce Rappaport Faculty of Medicine, Technion-Israel Institute of Technology, Haifa, Israel; ^4^Department of Neurology, Rambam Health Care Campus, Haifa, Israel

## Abstract

Ablative incisionless neurosurgery has become possible through advances in focused ultrasound and magnetic resonance imaging (MRI). The great advantage of MRI-guided focused ultrasound (MRgFUS) is that the ablation is performed through an intact skull without surgery. Here, we review the new modality of MRgFUS for treating tremor and enlighten the role of the anesthesiologist in the unique procedural setting of the MRI suite. During the MRgFUS process, the patients should be awake and are required to cooperate with the medical staff to allow assessment of tremor reduction and potential occurrence of adverse effects. In addition, the patient's head is immobilized inside the MRI tunnel for hours. This combination presents major challenges for the attending anesthesiologist, who is required to try to prevent pain and nausea and when present, to treat these symptoms. Anxiety, vertigo, and vomiting may occur during treatment and require urgent treatment. Here, we review the literature available on anesthetic management during the procedure and our own experience and provide recommendations based on our collected knowledge.

## 1. Introduction

Tremor is a common neurological disorder, most frequently observed in patients with essential tremor (ET) and Parkinson's disease (PD) [1–3]. In ET, the tremor is postural or kinetic, while in PD, it presents as rest tremor with postural and kinetic tremor appearing in a subset of patients. Tremor may vary from a mild degree of tremor causing social embarrassment to a severe disabling state. It may affect the quality of life by disrupting daily activities including eating, drinking, writing, and other functions that require controlled and accurate movement.

When tremor affects patients' lives, medications are warranted. Unfortunately, tremor is responsive to medications only in about 50% of patients [4]. When tremor is disabling and unresponsive to medication, neurosurgical intervention is proposed. Until recently, deep brain stimulation (DBS) was offered to most patients. In this surgery, electrode wires are implanted into specific basal nuclei and then connected to a subcutaneous battery-powered neurostimulator [5]. Adverse events of DBS are high, deterring patients from undergoing this treatment [5–7].

High-intensity focused ultrasound is a noninvasive technology. In this procedure, ultrasound waves are directed to converge in a precise target in the body, where they are absorbed by the tissue and converted to heat. Heating causes tissue ablation confined to the target, sparing neighboring tissue [8–11].

Magnetic resonance imaging- (MRI-) guided focused ultrasound (MRgFUS) using the Exablate® system (INSIGHTEC, Tirat Carmel, Israel) is a relatively new modality for tremor treatment that combines high-intensity focused ultrasound and MRI to accurately and noninvasively ablate the ventral-intermediate (VIM) nucleus of the thalamus [8–11]. The great advantage of MRgFUS is that the ablation is done on an intact skull without surgery [8–11]. The MRI allows treatment planning and continuous real-time monitoring, thus enabling adjustment of treatment parameters to optimize and personalize treatment outcomes. It has recently been approved by the Food and Drug Administration for treating refractory ET [12]. Since MRgFUS causes irreversible lesioning, the procedural accuracy is crucial. The permanent lesion is performed only after confirming the correct lesion location based on MR imaging and patient's feedback. Therefore, the patient should be awake during the MRgFUS procedure and is required to cooperate with the medical staff in order to allow assessment of tremor reduction and potential occurrence of adverse effects. As the temperature is gradually increased, the patient is repeatedly questioned regarding any unpleasant sensation such as headache, nausea, vertigo, pain, paresthesias, and claustrophobia, which are the most common complaints during the procedure. During the procedure, the patient's head is immobilized inside the MR tunnel presenting major challenges for the attending anesthesiologist.

Here, we review the literature available on anesthetic management during the procedure and our center's experience and provide recommendations based on our collected knowledge.

## 2. The Anesthesia Regimen during MRgFUS VIM Thalamotomy

### 2.1. Preoperative Evaluation

Careful patient selection is a major determinant of successful treatment. MRgFUS VIM thalamotomy is considered if patients have medication-refractory moderate-to-severe tremor [10, 11, 13]. In our center, patients are evaluated for MRgFUS by a multidisciplinary team that includes a movement disorders neurologist, a neurosurgeon, and an anesthesiologist. Factors which are taken into consideration are the patient's general physical condition, risk factors for bleeding or overheating of undesired tissue, contraindications for MRI, and other intracranial or cognitive pathology, as well as the severity of the functional disability and the expected improvement in quality of life following the treatment. Contraindications for treatment include brain tumors, unstable cardiac status, severe hypertension, current medical condition resulting in abnormal bleeding and/or coagulopathy, anticoagulant (e.g., warfarin) or antiplatelet (e.g., aspirin) therapy within 10 days of the planned procedure, inability or unwillingness to tolerate the required prolonged stationary supine position during treatment, claustrophobia, history of seizures within the past year, history of intracranial hemorrhage multiple strokes or stroke within the past 6 months, and standard contraindications for MR imaging. The ability of the patient to cooperate during the procedure is carefully assessed, since it may last for an average of 4 hours at the MRI suite with the head affixed to the MRI table. A clear description of the expected process is given to the patients to exclude to ascertain that there would be realistic expectations from the treatment outcome. Since MRgFUS is currently offered for unilateral control of tremor, the treated side is determined according to the patient's preference.

Three-dimensional CT scan is performed before the procedure to calculate the skull thickness, density, and skull density ratio (SDR). A low SDR, especially if below 0.35, reduces the chances of successful treatment and prolongs the procedure. A three-dimensional MRI is performed prior to the final decision on treatment to exclude brain findings, which are contraindications for MRgFUS [14].

Antitremor medications, taken by the patient, are tapered off days before the procedure according to their pharmacokinetic half-life to ensure that the most pronounced tremor will be evident and to assure that the tremor is ameliorated by the treatment. When possible, antihypertensive medication that may affect tremor, such as propranolol, is also discontinued, and if needed, a different antihypertensive is prescribed.

The patient is admitted in our center the evening before the procedure for convenience, but this is not mandatory. The standard preoperative fasting regimen is employed. Instructions are to avoid eating for 6 hours and drinking for 2 hours prior to the procedure.

### 2.2. Surgical and Anesthetic Techniques

The MRgFUS VIM thalamotomy is performed as a single-day case and usually lasts between 3.5 and 5 hours. On the day of the procedure, an intravenous line is inserted as well as a urinary catheter in women and a Penrose drain tube in men. The patient wears silastic pressure stockings to prevent potential deep vein thrombosis [9].

The patient's head is completely shaved. The stereotactic frame crown (CRW; Integra, Plainsboro, NJ) is positioned on the skull with the patient sitting up, facing the surgeon. The projected pin/screw entry sites are marked with a marking pen, and thereafter, the frame is removed. Local anesthesia is injected in the predetermined spots. The pins are then inserted and tightened. An elastic silicone membrane to seal the circulating degassed water comprising the interface between the transducer and the target is attached to the patient's head. The membrane is cut to fit the patient's head diameter to prevent unnecessary pressure on the skull. The patient then walks to the MRI table. The head frame is firmly attached to the MRI table. The patient is positioned with the stereotactic frame in the MRI tunnel so that the head is immobilized. From this point on, the patient cannot be released without unlocking the frame from the table. At this stage, a planning MR scan is performed in 3 axes. These MR scans are fused with preprocedural 3-axis MRI, CT, and early mockup planning to accurately determine the treatment target. Thereafter, cold degassed water at 16°C fills the gap between the silicone membrane and the MRgFUS helmet.

Sonication refers to the administration of energy to the target using focused ultrasound beams. The ultrasonic beams are directed to the VIM thalamus through the helmet-like focused ultrasound transducer (Exablate 2000; InSightec Inc., Haifa, Israel). Multiple sonications are administered gradually, increasing the power and temperature to ascertain the procedure's safety [10, 11]. Each sonication lasts between 10 and 39 seconds. The energy heats the tissue. The temperature is measured by MR thermometry [15]. After each sonication, the treatment table is drawn out of the MRI tunnel, and the patient is assessed for adverse effects and improvement in tremor.

The treatment is divided into several stages: in the first stage, sonications are delivered at a very low energy to confirm that the focus is in the selected target. If required, the sonication focus is adjusted. The temperature at this stage typically reaches 41–46°C. At these temperatures, some patients report discomfort due to lying supine for a relatively prolonged period of time (∼1–1.5 hours). The second stage involves sonications with gradually increasing energy to achieve a temporary effect on tremor and to confirm the absence of adverse effects. Target coordinates are repositioned as necessary according to the clinical status, the patients' feedback, and adverse effects, if any. Typically, temperatures at this stage reach 46–50°C. As the temperature is increased, the patient may experience unpleasant transient sensations from the sonication due to the heat that develops in the brain or the skull. This may be manifested by symptoms including lip or finger paresthesias, headache, vertigo, nausea, and even vomiting. The third stage is reached once the selected target site clearly shows a reduction in tremor and no adverse effects. The temperature is then increased to induce a permanent lesion. This is referred to as the ablative stage, and it is expressed by a gradual increase of the total ablative energy, either via augmenting sonication intensity or by extending sonication duration. This causes temperature elevation in the tissue to at least 57°C. Heating any tissue for one second at 57°C (or with an equivalent thermal dose) denatures proteins. The area of tissue exposed to the temperature and the length of exposure to this heat define the extent of the lesion created by the ultrasound beams [16]. Repeated sonications are performed with the maximal energy to achieve the long-lasting effect. The sonication phase is concluded once adequate control of tremor is achieved, with temperatures reaching no more than 60°C.

### 2.3. Anesthetic Considerations

Since the anesthetic management of patients undergoing MRgFUS treatment has unique aspects, the anesthesiology team should evaluate the patients before the procedure. The anesthesiologist's role in MRgFUS is to provide the patients with adequate comfort, monitor the patients throughout the procedure, and diagnose and treat complications that may arise [8, 11].

The patient is frequently anxious before the procedure, but premedications of the benzodiazepine family of drugs and other GABA agonists that could interfere with cooperation and tremor interpretation must be avoided. Anesthetic agents that may influence tremor or the ability to communicate with the team should also be avoided. The team should try to relieve the tension with words of reassurance and create a good atmosphere.

About 30 minutes after applying EMLA® (lidocaine/prilocaine) cream and after administering local anesthesia containing a mixture of lidocaine 2% and bupivacaine 0.5%, the head frame is affixed to the patient's head (Table 1) A silicone frame is then affixed to the patient's head. Once positioned in the MRI tunnel, the patient is required to lie still for 3–5 hours while awake and immobilized. This may stimulate discomfort, claustrophobia, isolation, and anxiety. Therefore, the patient must be well prepared and informed beforehand, understand the various stages of the planned process, and be motivated to be cooperative during the entire procedure for the completion of the procedure. The patient should be told that, in case of any significant discomfort or problem, the procedure can be stopped at any moment. It is necessary to emphasize to the patient that the anesthesiologist will always be close by and available as needed. A family member or a friend waits outside the MRI suite in case the patient feels unsettled. Since all anxiolytics reduce tremor, we refrain from using them throughout the procedure.

Sonication exertion might cause nausea and even vomiting [9–11]. Vomiting while lying supine with the head firmly fixed to the transducer may lead to aspiration or choking. Our protocol is to administer intravenously antiemetics and analgesics 30 minutes prior to the procedure, providing no known contraindications and allergies exist. For antiemesis, we administer granisetron 3 mg before treatment or ondansetron 4 mg before treatment and an additional 4 mg during treatment or, in ET patients, metoclopramide hydrochloride 10 mg. Dexamethasone as an antiemetic is also used unless the patient's fasting blood sugar is above 120 mg/dl.

Nausea may not respond to intravenous ondansetron or metoclopramide. When the maximal dose of these medications was achieved and the patient still complained of nausea, we found that a few drops of lemon juice were helpful. If vomiting occurs, it must be treated immediately. In such an emergency case, the water around the patients' head can be emptied within a couple of seconds. Yet, the anesthesiologist should be prepared to start treating the patient in the supine position with the frame still on the head, since disconnecting the frame from the bed and unscrewing the frame may take a few minutes. In case that intubation is required, the anesthesiologist should urgently call for help.

The blood sugar level is checked before the procedure. An oxygen nasal prong is attached. The patient is given an emergency stop button that when pressed, shuts down the sonication and calls the team.

The anesthesiologist should prepare an MRI-compatible laryngoscope, endotracheal tubes, laryngeal mask airway, and suction. During the procedure, the anesthesiologist should monitor the patient with a pulse oximeter, noninvasive blood pressure cuff, electrocardiograph (ECG), and end-tidal CO_2_ with a designated nasal prong. The use of the basic monitoring equipment is our standard policy for each monitored anesthesia care (MAC) procedure. During the procedure, events such as desaturation, vasovagal reaction, and others may occur, although they are rare. Basic monitoring could help in recognizing them and taking measures if required.

The anesthesiologist can remain inside or outside the MRI room but must continuously monitor and hear the patient and the alarms. Each time, the MRI table should be pulled out during the procedure and the patient should be asked about possible discomfort, headache, nausea, and itch and checked for the severity of tremor. The most common procedure-related complaints are pressure exerted on the head by the bolts connecting the stereotactic frame to the skull and form the silicon membrane pressing on the skull [9–11]. When present, these complaints should be treated, since they may last throughout the procedure. The patients may also complain about headache that appears only during sonications. This type of headache can be severe, and in our experience, it may lead to discontinuation of the procedure. Treatment of such an headache includes intravenous paracetamol 1 g, dipyrone 1 g, or ketorolac 30 mg.

Another common complaint is backache due to prolonged immobility. We have used a specialized cushion that flexes the legs and reduces pressure on the lower back. The patient may also receive intravenous paracetamol to relieve this ache. For neck pain prevention, a small cushion is used as well.

## 3. Conclusions

MRgFUS is a new technology that presents unique challenges since the patient should be awake throughout the procedure and affixed to the treatment table inside the MR tunnel. Successful results can only be achieved with a multidisciplinary team that is familiar with the procedure. The anesthesiologist has an important role in this team.

## Figures and Tables

**Table 1:** Anesthetic regimen in MRgFUS.

Indication	Medication/treatment	Special considerations
Pain prevention	Intravenous paracetamol 1 g with dexamethasone 12 mg, unless fasting blood sugar >120 mg/dl	One hour before the procedure
Nausea prevention	Intravenous granisetron 3 mg or ondansetron 4 mg, or metoclopramide hydrochloride 10 mg in essential tremor patients. If protracted, dexamethasone 12 mg, unless fasting blood >120 mg/dl	One hour before treatment
Pain from pressure of the silicone membrane	EMLA lubrication of the skull before fitting the silicone membrane	30 minutes before membrane placement
Pain from pins of the head frame	Locally injected lidocaine 2% and bupivacaine 0.5%, at each pin site	Immediately before pin insertion
Backache	Usually not needed. May administer intravenous paracetamol 1 g, dipyrone 1 g, or ketorolac 30 mg	Use of specialized cushion that flexes legs and reduces pressure on the lower back
Neck pain	Usually not needed. May administer intravenous paracetamol 1 g, dipyrone 1 g, or ketorolac 30 mg	Use of small cushion below the neck
Anxiety	None	Employing nonpharmacologic strategies
Nausea during treatment	Intravenous ondansetron 4 mg	A few drops of lemon juice may be tried
Vomiting	Suction	Urgent call for help
Vertigo	None	Reassurance since transient
Miscellaneous	Oxygen 2-3 l/min via the nasal prong. Emergency stop button that shuts down sonication and calls for help is given to the patient	—

EMLA: eutectic mixture of local anesthetics; mg: milligram; dl: deciliter; ml: milliliter; l: liter; min: minute.
